# Graphene-Based Two-Dimensional Mesoporous Materials: Synthesis and Electrochemical Energy Storage Applications

**DOI:** 10.3390/ma14102597

**Published:** 2021-05-16

**Authors:** Jongyoon Park, Jiyun Lee, Seongseop Kim, Jongkook Hwang

**Affiliations:** 1 Department of Energy Systems Research, Ajou University, 206 Worldcup-ro Yeongtong-gu, Suwon 16499, Korea; whddbs8294@ajou.ac.kr (J.P.); jiyun0408@ajou.ac.kr (J.L.); 2 Department of Chemical and Biomolecular Engineering, Korea Advanced Institute of Science and Technology (KAIST), 291 Daehak-ro, Daejeon 34141, Korea; seongseopkim@kaist.ac.kr; 3 Department of Chemical Engineering, Ajou University, Worldcupro 206, Suwon 16499, Korea

**Keywords:** two dimensional materials, graphene, mesoporous materials, supercapacitor, lithium-ion batteries

## Abstract

Graphene (G)-based two dimensional (2D) mesoporous materials combine the advantages of G, ultrathin 2D morphology, and mesoporous structures, greatly contributing to the improvement of power and energy densities of energy storage devices. Despite considerable research progress made in the past decade, a complete overview of G-based 2D mesoporous materials has not yet been provided. In this review, we summarize the synthesis strategies for G-based 2D mesoporous materials and their applications in supercapacitors (SCs) and lithium-ion batteries (LIBs). The general aspect of synthesis procedures and underlying mechanisms are discussed in detail. The structural and compositional advantages of G-based 2D mesoporous materials as electrodes for SCs and LIBs are highlighted. We provide our perspective on the opportunities and challenges for development of G-based 2D mesoporous materials. Therefore, we believe that this review will offer fruitful guidance for fabricating G-based 2D mesoporous materials as well as the other types of 2D heterostructures for electrochemical energy storage applications.

## 1. Introduction

Two-dimensional (2D) nanomaterials are an emerging class of anisotropic sheet-like materials with the lateral dimensions ranging from hundreds of nanometers to several micrometers and the thickness of few-atoms <5 nm [1,2]. Since the first exfoliation of single-layer graphene from graphite using Scotch tape in 2004 [3], a wide variety of 2D materials have been investigated intensively, such as graphene [4], hexagonal boron nitride [5], graphitic carbon nitride [6], transitional metal dichalcogenides [7], layered double hydroxides [8], MXene [9], etc. Ultrathin 2D materials can exhibit unprecedented physical, chemical and optical properties that are otherwise difficult to realize. In particular, graphene (G), a defect-free 2D carbon monolayer with a hexagonal lattice, exhibits excellent electrical conductivity (~2000 S cm^−1^) and thermal conductivity (~5000 W m^−1^ K^−1^), high Young’s modulus (~1 TPa), and a large theoretical surface area (2630 m^2^ g^−1^) [10,11,12]. Because of these unique properties, G and G-based materials have attracted great research interest from basic science to various applications [13,14].

Porous materials are classified as microporous (<2 nm), mesoporous (2–50 nm) and macroporous (>50 nm) materials according to the definition of the International Union of Pure and Applied Chemistry (IUPAC). Development of ordered mesoporous aluminosilicate in 1992 [15] sparked great research interest in mesoporous materials with well-defined structures and morphologies. Ordered mesoporous materials show outstanding properties including uniform pore size, periodic pore structures, high specific surface area and large pore volume. In addition, they have been used as versatile hosts for stabilizing secondary guest species, allowing the synthesis of a wide variety of hybrid mesoporous composites suitable for diverse applications such as catalysis, sensors, and energy storage and conversion [16,17,18,19,20]. Previous research efforts have found that the size, geometry, distribution of pores [21,22,23,24,25] as well as particle size and morphology [26,27,28,29,30,31] have a significant impact on the final performance of the mesoporous materials. In this regard, the development of efficient strategies for controlling nano- and microstructures and morphologies has long been sought in the field of porous materials science.

Therefore, it is natural to fabricate 2D mesoporous materials/G heterostructures that combine the advantages of G, ultrathin 2D morphology, and periodic mesoporous structures. In particular, G-based 2D mesoporous materials have shown great potential as high-performance electrode materials in various energy storage applications such as supercapacitors (SCs) and lithium-ion batteries (LIBs) [10,11,12,32,33,34,35]. Each factor shows great synergy, contributing to the increase of power and energy densities of energy storage devices due to the following advantages: (i) G works as a robust and flexible 2D substrate for supporting mesoporous materials, opening up the possibilities to fabricate freestanding flexible electrodes [36]. G also acts as conductive matrix boosting electrical conductivity and rate capability of the electrodes. Thus, the use of conductive additives and/or current collectors can be minimized, which can significantly increase the overall energy density. (ii) Ultrathin 2D morphology with large lateral size and atomic thickness give rise to high theoretical specific surface area, which is attractive in the surface-driven charge storages in SCs [12]. Two-dimensional materials usually have high processability for fabrication of freestanding electrodes with high packing density [1,37,38]. (iii) Mesoporous structures increase the accessible surface area for electrosorption and serve as ion highways allowing for fast ion transport [39,40]. Mesopores can further host guest species with high theoretical capacity. In addition, uniform pores can effectively buffer the volume changes caused by repeated ion insertion/de-insertion, thereby increasing the cycle stability of the LIBs [41,42,43,44]. Furthermore, by depositing mesoporous material on G, it is possible to decrease the degree of G aggregation and restacking that significantly deteriorates the unique properties of the single-layer G [4].

Several reviews on 2D materials [1,2,45,46,47], G [10,11,32,33,48] and mesoporous materials [4,17,20,49,50,51,52,53] have been published; however, none has provided a complete overview of G-based 2D mesoporous materials. Hence, in this review, we comprehensively summarize the synthesis of G-based 2D mesoporous materials and their application in SCs and LIBs. First, we classified the synthesis strategies for G-based 2D mesoporous materials into four classes: soft templating, hard templating, template-free and top-down methods. Each section was then subdivided according to the composition of mesoporous materials: silica, carbon, metal oxide and others. Next, we introduced the use of G-based 2D mesoporous materials as advanced electrodes in SCs and LIBs. Finally, we provided our perspective on the opportunities and challenges for development of G-based 2D mesoporous materials in the field of energy storage devices.

## 2. Synthesis Strategies

Direct growth of mesoporous materials on G would be the simplest way to prepare 2D mesoporous materials/G composites. However, G is rarely used to prepare these composite materials due to the difficulty of mass production, high production costs and low dispersibility in most solvents [12]. Instead of G, graphene oxide (GO) was employed as a 2D substrate for controlled growth of mesoporous materials. GO is produced as a single-layer nanosheet by oxidation of pristine graphite, known as the Hummers method [54]. GO is a defective form of G and possesses hydrophilic functional moieties such as carboxyl, hydroxyl and phenol groups. As a result, GO disperses more easily in a wide range of solvents and is friendly to the sol-gel chemistry [49].

Therefore, in a typical synthesis, GO is mainly utilized as a morphology-directing substrate that directs the growth of secondary mesoporous layers, often resulting in sandwich-like hybrid nanosheets. However, it should be mentioned that GO must be reduced to restore the characteristic of G. Various chemical, thermal and electrochemical reduction processes have been applied to convert GO to G or reduced graphene oxide (rGO), increasing overall thermal/electrical conductivity of the composite materials.

Controlling the interaction between GO, templates, and inorganic precursors plays decisive role in preparation of G-based 2D mesoporous materials. Thus, the surface functionalities of GO, types of soft template, pH, concentration, and temperature were carefully adjusted to direct the nucleation and growth of inorganic mesoporous materials on GO surface in a controlled manner. To date, a wide range of G-based 2D mesoporous materials such as silica, carbon, metal oxide and metals have been fabricated through soft templating (Section 2.1), hard templating (Section 2.2), template-free (Section 2.3) and top-down (Section 2.4) methods. We have categorized the reported literature thoroughly in Table 1 with respect to synthetic strategies, materials composition, physicochemical properties, and applications. The general aspect of synthetic strategies, synthetic procedures and underlying mechanisms were provided in detail.

### 2.1. Soft Templating Method

The combination of GO and soft templates such as surfactant and block copolymer (BCP) is the method of the first choice for controllable synthesis of G-based 2D mesoporous materials. This approach has high flexibility in controlling the pore size and structures of mesoporous materials because of tunable composition, molecular architecture and the molar mass of soft templates. Cationic surfactants (cetyltrimethyl ammonium bromide (CTAB), cetyltrimethyl ammonium chloride (CTACl)), anionic surfactant (sodium dodecyl sulfate (SDS)), commercial pluronic BCPs (P123, F127), and lab-made BCPs (poly(styrene)-*b*-poly(2-vinylpyridine)-*b*-poly(ethylene oxide) (PS-*b*-P2VP-*b*-PEO), poly(styrene)-*b*-poly(ethylene oxide) (PS-*b*-PEO) have been utilized as structure-directing agents. Soft templates leave mesopores after removal by solvent extraction or thermal pyrolysis. They adsorb on the GO surface and increase the interaction between GO and other precursors (silicates, carbon, metal oxides), thereby facilitating the nucleation and growth of inorganic mesoporous material onto GO.

#### 2.1.1. Mesoporous Silica/Grapheme

**Figure 1:**
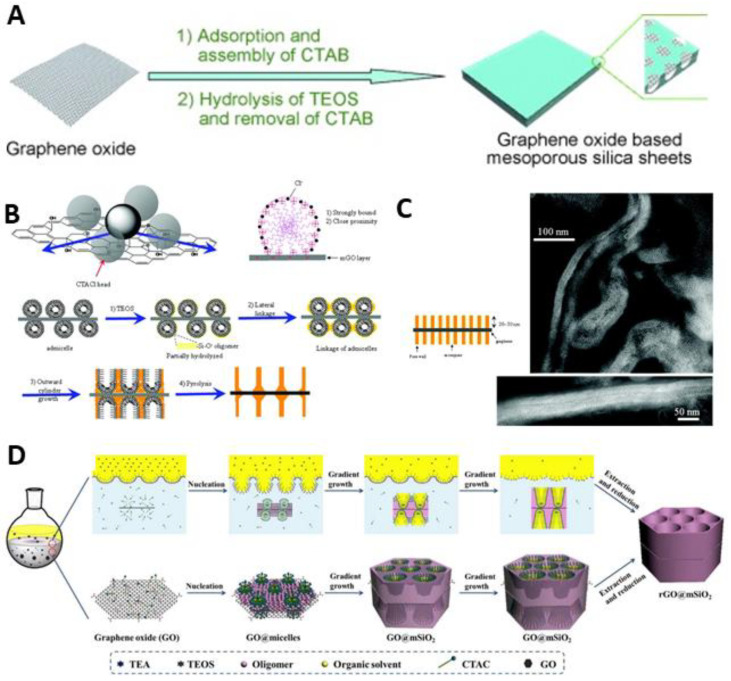
(**A**) Scheme of the fabrication process of mSiO_2_/G. Reprinted with permission from Ref. [55], Copyright (2010) John Wiley and Sons. (**B**) Scheme of a CTACl head group, an admicelle on mGO surface and proposed mode of formation of mSiO_2_/G sandwich structure with vertically oriented channels. (**C**) Microtome scanning transmission electron microscopy (STEM) image of mSiO_2_/G with vertically oriented channels prepared at pH 11.7. (**B**,**C**) Reprinted with permission from Ref. [56], Copyright (2010) American Chemical Society. (**D**) Scheme of the synthesis process of the mSiO_2_/G nanosheets by an oil-water biphase stratification approach. Reprinted with permission from Ref. [57], Copyright (2015) American Chemical Society.

Since the first report in 1992 [15], ordered mesoporous silicates (mSiO_2_) have been the subject of intensive research and their synthesis protocols have been well-established in terms of controlling pore size, structures and morphologies [24,114,115,116,117]. Therefore, it is natural to first integrate mSiO_2_ with G to prepare 2D mesoporous nanosheets. In 2010, Müllen and coworkers reported the synthesis of hexagonal mSiO_2_/G composites with disordered mesoporous structure, using CTAB as a structure-directing agent (Figure 1A) [55]. Cationic CTAB electrostatically adsorbed and self-assembled onto the surface of negatively charged single layer GO. During this process, the cationic surfactant prevented an aggregation between GO and inorganic materials and simultaneously functioned as a template for controlled nucleation and growth of mSiO_2_. After thermal treatment of these sheets at 800 °C under inert atmosphere, GO was reduced to individual G without aggregation, forming 2D mSiO_2_/G composites.

Wang et al. reported another example of a mSiO_2_/G sandwich structure with vertically oriented channels by using the cationic CTACl. They thoroughly investigated the effects of pH, the amount of silica precursor, the ratio of CTACl to GO and the temperature on structure formation, and thus established synthesis conditions for unique structural nanocomposites whose channels grew perpendicularly on both surfaces of the GO platelets. The co-assembly of CTA with the silica and subsequent polymerization resulted in a structural transformation of spherical admicelles to cylindrical ones growing perpendicular to the surface of GO (Figure 1B,C) [56]. Interestingly, Liu et al. developed a new oil-water biphase stratification approach to synthesize size-tunable and vertical funneling mesochannels, owing to the pore-expanding effect of organic solvent. Tetraethyl orthosilicate (TEOS) in the hydrophobic oil phase gradually diffused to the biphase oil-water interface, making TEOS hydrolyzed and hydrophilic enough to attach to GO in the water phase (Figure 1D) [57].

Furthermore, mSiO_2_ acts as the porous host material for guest species. It stabilizes and disperses guest species such as polyethyleneimine (PEI), metal nanoparticles (NPs) and drugs, providing additional functions to the G nanosheets. For example, Yang et al. developed sandwich-like PEI@mSiO_2_/G sheets as superior CO_2_ adsorbent. mSiO_2_ stabilized a large amount of PEI (60 wt%) within the mesopores while G offers high thermal conductivity and stability. The resulting PEI@mSiO_2_/G had efficient CO_2_ diffusion and adsorption ability and fast heat transfer, exhibiting higher CO_2_ adsorption capacity and cycle stability than PEI and PEI/m-SiO_2_ sheets [61].

In this regard, Zhang and coworkers proved that the sandwich method could also be applied for preparation of robust heterogeneous catalysts, by encapsulating small metal NPs in-between the mSiO_2_ layer and the G nanosheets. The high catalytic performance in various reactions was attributed not only to the large surface area of G, but also the mSiO_2_ layers which stabilize the ultrafine Pt NPs and prevent aggregation among the G nanosheets [60]. On the other hand, the mSiO_2_/G can be also used as a powerful carrier for the delivery of antitumor drugs by loading a targeting peptide-modified mSiO_2_/G. The 2D mSiO_2_/G architecture and peptide modification played a decisive role in enhancing the accumulation of nanocomposite within the cancer cell significantly [59].

Although the use of cationic surfactants (e.g., CTAB, CTACl) enables the preparation of various mSiO_2_/G, the short chain length (~2 nm) of surfactants imposes significant limitations on the fabrication of large pore ordered mesostructures. Typically, the GO surface is covered with negatively charged functional groups that are ~1 nm thick, similar to the chain length of the surfactant. The bulky surface groups of GO would interact with surfactant too strongly and even hinder the self-assembly of CTAB-silicates, thereby often leading to disordered mesoporous structures. This may also explain why surfactant-directed preparation of mSiO_2_/G is highly sensitive to the synthetic conditions such as pH, temperature and reagent concentration. In this respect, Lee et al. used a non-ionic triblock copolymer (pluronic P123) instead of an ionic surfactant [58]. It was believed that P123 diminished undesired surfactant-surfactant interaction and surfactant-surface interaction and promoted the formation of a highly ordered mSiO_2_/G nanocomposites. P123 worked as a structure directing agent for formation of bicontinuous KIT-6 silica and as a cross-linking agent that bridged KIT-6 and G together.

#### 2.1.2. Mesoporous Carbon/Grapheme

**Figure 2:**
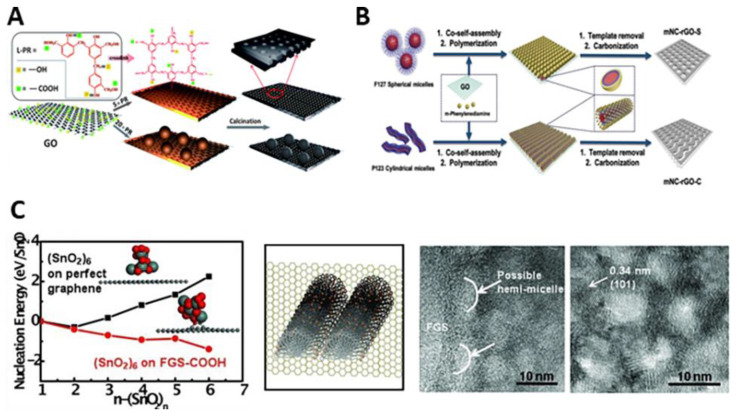
(**A**) Scheme of the preparation of mesoporous carbon/graphene (mC/G). Reprinted with permission from Ref. [62], Copyright (2013) The Royal Society of Chemistry. (**B**) Scheme of the fabrication of N-doped mC/G nanosheets with spherical or cylindrical pores through the interfacial self-assembly of Pluronic block copolymers in solution. Reprinted with permission from Ref. [63], Copyright (2019) John Wiley and Sons. (**C**) Density functional theory (DFT) calculation of SnO_2_ nucleation on pristine G and on functionalized G sheets with a carboxylic group linking to the defect site, G template with the formation of ordered surfactant micelle structures, and transmission electron microscopy (TEM) images showing possible hemi-micelles at the interfaces and the crystalline mTiO_2_/G. Reprinted with permission from Ref. [69], Copyright (2012) John Wiley and Sons.

The soft templating method is simple and versatile, allowing mesoporous carbon (mC) to be grown directly on the GO surface. In 2013, Xue and coworkers reported direct preparation of graphene-based mesoporous carbon(mC/G) using low molecular weight phenolic resol as a carbon precursor and F127 as a soft template (Figure 2A) [62]. F127 was added to an aqueous solution of prepolymerized resol to form resol@F127 composite micelles. The addition of GO to the solution induced the composite micelles to self-assemble onto the GO surface through hydrogen bonding (micelles@GO). After hydrothermal treatment at 180 °C, the micelles were solidified through thermal polymerization of resol. Interestingly, as the mass ratio of micelles to GO increases from 5 to 20, the carbon morphology transformed from thin-layers to solid-spheres. Carbonization at 800 °C finally yielded the mC-layer/G or mC-sphere/G. Furthermore, pore structures can be engineered by using Pluronic BCPs with different compositions. Hou et al. demonstrated that F127 or P123 co-assembled with phenylenediamine (N-containing carbon precursor) to form spherical or cylindrical composite micelles on the G surface, respectively (Figure 2B) [63]. After polymerization of phenylenediamine and pyrolysis at 800 °C, N-doped mC/G with similar pore size (~8 nm in diameter), high specific surface area (~420 m^2^ g^−1^) and N contents (2.6 wt%) were produced.

Lab-made amphiphilic block copolymers with various chemical compositions and molecular weight such as (PS-*b*-P2VP-*b*-PEO) and (PS-*b*-PEO) can provide new opportunities for synthesis of large-pore sized mC/G (>10 nm) and diverse pore geometries [51,118]. For instance, Yamauchi and coworkers reported assembly of the composite micelles of melamine-formaldehyde resin and PS-*b*-P2VP-*b*-PEO on the surface of GO [64]. After carbonization at 900 °C, the monolayered solid nanospheres were converted into hollow nanospheres with an external diameter of ~28 nm and a hollow core of ~20 nm, as well as a high surface area (968.3 m^2^ g^−1^) and high nitrogen content (6.5 at%). Similarly, Mai and coworkers demonstrated the co-assembly of PS-*b*-PEO, pyrrole monomers and molybdate ions on GO surfaces to prepare N-doped mC-MO_2_C/rGO [65]. The same group also prepared mC-Fe_2_O_3_/rGO with tunable mesopores (12–23 nm) by changing the length of PS block of PS-*b*-PEO templates [66].

#### 2.1.3. Mesoporous Metal Oxide and Others/Graphene

Mesoporous metal oxide (MO)/G can be synthesized by the same approach reported to create mSiO_2_/G and mC/G (see Section 2.1.1 and Section 2.1.2) [55,62]. Yang group obtained mSnO_2_/G through a cooperative assembly of CTAB-SnO_2_ on GO layer [67]. Under highly basic conditions, GO layers in solution were negatively charged and adsorbed the CTA^+^ ions through electrostatic interactions. Subsequent addition of Sn^4+^ resulted in a rapid reaction with OH^−^, forming negatively charged Sn(OH)_x_ species at the interface of the micelles. After solvothermal reaction and removal of CTAB surfactants through washing process, mSnO_2_/G with high specific surface area (251.7 m^2^ g^−1^) and pore size (3.8 nm) was produced.

Wang et al. used anionic sulfate surfactants, SDS, to increase the interactions between GO and the TiO_2_ materials. The surfactants adsorbed onto GO through the hydrophobic tails, making GO sheets highly dispersed and interact with the TiO_2_ precursor through the hydrophilic head groups. Thus, SDS surfactants stabilized GO sheets in aqueous solutions and facilitated the controlled growth of nanocrystalline TiO_2_ on GO.

A wide range of ordered mesoporous materials have been synthesized by evaporation-induced self-assembly (EISA) methods [119]. Through EISA, the soft template and various inorganic building block can co-assemble to form organic–inorganic hybrid composites [40]. Inspired by this, Li et al. synthesized SnO_2_/G, ZrO_2_/G and TiO_2_/G by combination of EISA and GO (Figure 2C) [69]. They studied the nucleation and self-assembly processes both theoretically and experimentally. Density functional theory calculation showed that nucleation of MO clusters was energetically favorable at the functional groups of G sheets. Surfactant molecules also lowered the nucleation energy and promoted the nucleation of MOs on G. Considering these factors, the authors dispersed functionalized G to a solution of P123, acid and MO precursor. Under acidic condition, P123 molecules were likely absorbed on functionalized G sheets surface as hemi-micelles. During EISA, the micelle and hydrolyzed-metal precursor were gradually co-assembled on functionalized G surface. After annealing at 400 °C, the as-made amorphous hybrid converted to crystalline MO/G with surface area of ~150 m^2^ g^−1^ and pore size of 6–10 nm.

Instead of in situ growth of inorganics on GO, alternatively, Yamauchi and coworkers hybridized the pre-synthesized mesoporous tin phosphate (mSnPi) flakes with GO [70]. mSnPi was prepared by using F127, H_3_PO_4_ and SnCl_4_ in a mixed solution of ethanol, water and HF. Subsequently mSnPi was added to GO suspension at basic pH~10, generating mSnPi wrapped and/or covered with thin layers of GO. The resulting mSnPi/GO had high specific surface area (170 m^2^ g^−1^) and uniform mesopores (7.4 nm).

### 2.2. Hard Templating Method

Preformed mSiO_2_ and colloidal crystals have been widely utilized as hard templates for preparation of a variety of inverse-replicated mesoporous carbon, metal oxides and others [120,121]. The hard templating method is particularly useful in preparation of highly crystalline materials (e.g., metal oxides) that require high temperature pyrolysis. Hard template functions as a rigid support that keeps the mesostructures intact during crystallization at high temperature > 500 °C. In addition, the use of mSiO_2_/G as a hard template can solve the problem that carbon (or metal oxide) NPs are randomly anchored to the nanosheet. Thus, mSiO_2_/G has been widely used as 2D hard templates to G-based mesoporous materials.

#### 2.2.1. Mesoporous Carbon/Grapheme

**Figure 3:**
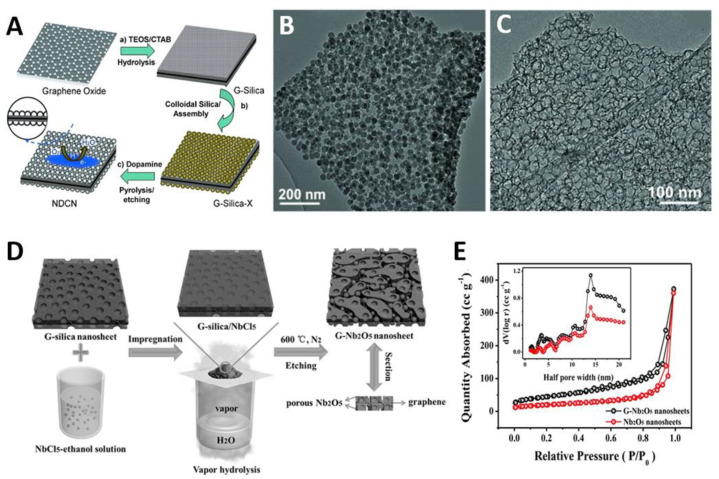
(**A**) Scheme of the synthesis of N-doped mC/G. (**B**,**C**) TEM images of mSiO_2_/G and inverse-replicated N-doped mC/G. (**A**–**C**) Reprinted with permission from Ref. [72], Copyright (2014) John Wiley and Sons. (**D**) Scheme of the preparation of Nb_2_O_5_/G nanosheets. (**E**) N_2_ adsorption/desorption isotherms of Nb_2_O_5_ and Nb_2_O_5_/G nanosheets. (**D**,**E**) Reprinted with permission from Ref. [77], Copyright (2016) John Wiley and Sons.

The Müllen group prepared mSiO_2_/G through a cooperative assembly of CTAB-silicates on GO (see Section 2.1.1) and then used it as the hard template to fabricate disordered mC/G nanosheets [55]. They further extended this strategy to obtain mesoporous carbon nitride (mCN)/G [71]. Ethylenediamine and carbon tetrachloride were impregnated into the pores of mSiO_2_/G, and converted to mCN after polymerization, pyrolysis and HF etching. However, the CTAB-derived mesopores are inherently smaller than 2 nm because of the short chain length of CTAB. To obtain large-pore sized mC/G nanosheets, Wei et al. further deposited colloidal SiO_2_ NPs of different sizes (7–22 nm) on CTAB-templated mSiO_2_/G [72]. Since the surface charge of both mSiO_2_/G and colloidal SiO_2_ are negative over the whole pH range, mSiO_2_/G should be functionalized by positively-charged poly(diallyl dimethylammonium chloride) (PDDA). After PDDA functionalization, the negatively charged colloidal SiO_2_ electrostatically assembled on the surface of mSiO_2_/G in a controlled manner. Subsequent polydopamine (PDA) coating, pyrolysis and silica removal led to N-doped mC/G with the uniform and tunable mesopores (7–22 nm) (Figure 3A–C). Along these lines, Kim and coworkers also synthesized the highly ordered mC/G with slightly larger mesopores (4 nm) using KIT-6/G as a hard template [74].

Without the use of either preformed SiO_2_ hard template or micelle template, Yu and coworkers synthesized N-doped mC/G through the direct cooperative assembly of silica aggregates and 3-aminophenol/formaldehyde (APF) resin on the surface of GO [73]. It was found that balancing the polymerization rate of TEOS and APF was the key to the successful cooperative assembly. Since TEOS has a relatively faster polymerization rate than APF, the additional APF polymerization catalyst, ethylenediamine (EDA), was necessary. EDA was adsorbed on the negatively charged silica surface and initiated the polymerization of APF. Absence of EDA weakened interaction between silica and APF oligomers, resulting in non-uniform APF deposition on the GO surface. Notably, the single-layered mC/G nanosheets with tunable mesopores (4.7–13.9 nm) were obtained by varying the amount of TEOS.

#### 2.2.2. Mesoporous Metal Oxide and Others/Graphene

As a typical example, the Müllen group prepared mTiO_2_/G nanosheets using mSiO_2_/G as the hard template and (NH_4_)_2_TiF_6_ as a titania precursor in a sol-gel process [75]. The resulting TiO_2_/G nanosheets exhibited not only mesoporous structure with a surface area of 202 m^2^ g^−1^ but also enhanced electrical conductivity. This group also demonstrated an essential role of the G layer for the improvement of the cycle performance by comparing TiO_2_/G nanosheets with TiO_2_ nanosheets without G. Similarly, Wang’s group fabricated carbon-coated mSnO_2_/G/mSnO_2_ nanosheets with high specific surface area (385 m^2^ g^−1^) and uniform mesopore size (3–4 nm) [76].

Wang et al. used mSiO_2_/G nanosheets as templates and niobium chloride as a Nb source [77]. An ethanol solution of NbCl_5_ was impregnated into the pores of CTAB-derived mSiO_2_/G template, followed by hydrolysis via water vapor and calcination at 600 °C under nitrogen atmosphere. After the removal of silica template, partially single-crystalline Nb_2_O_5_/G nanosheets with a high specific surface area of 158 m^2^ g^−1^ and the pore size of 3 to 20 nm were prepared (Figure 3D,E). The pores of 3 nm were generated from the replication of mSiO_2_/G, while pores larger than 3 nm arose during the crystallization process of Nb_2_O_5_, which is related to the loss of water.

### 2.3. Template-Free Method

A template-free approach has also been proposed as a relatively inexpensive and straightforward method for synthesizing G-based 2D mesoporous materials. The template-free approach usually relies on bottom-up aggregation of inorganic precursors or NPs on the GO surface. The mesopores originated from the inter- or intra-particle voids of the materials. Therefore, the template free approach generally lacks ability to control the pore size and structures, often yielding disordered random pores only.

#### 2.3.1. Mesoporous Carbon/Grapheme

**Figure 4:**
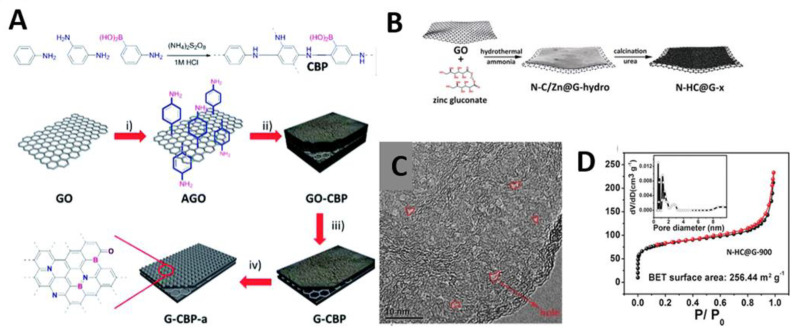
(**A**) Scheme of the preparation of N-doped mC/G and B/N-co-doped mC/G nanosheets. Licensed under CC-BY from Ref. [84]. (**B**) Scheme of the fabrication of ultrathin N-doped holey carbon layer on GO. (**C**) High-resolution TEM (HRTEM) image of N-doped holey carbon layer on GO with red cycles showing the nanoholes formed. (**D**) N_2_ adsorption/desorption isotherms of N-doped holey carbon layer on GO. (**B**–**D**) Reprinted with permission from Ref. [80], Copyright (2018) John Wiley and Sons.

GO has been widely used as 2D morphology-direct substrate for preparation of 2D porous carbon. Various carbon sources such as resorcinol-formaldehyde, polydopamine, polyaniline, melamine-dialdehydes were coated on a GO substrate, followed by carbonization at high temperature > 600 °C. The resulting carbon nanosheets are usually microporous but sometimes become mesoporous depending on the types of carbon sources, carbonization temperature or the thickness of nanosheets. The Li and Lu group prepared microporous C/G nanosheets using amphoteric asparagine as a bridging molecule that connected the GO and the resorcinol-formaldehyde resin. Positively charged asparagine adsorbed on negatively charged GO through strong electrostatic interaction, and then induced in situ polymerization of resorcinol-formaldehyde resin [82,83]. The Qiao group developed template-free growth of polydopamine (PDA) with controllable thickness on a GO substrate [78]. In a mixture of DA and GO in phosphate-buffered saline solution (pH 8.5), DA self-polymerized on the surface of GO. PDA/GO hybrid with thickness of 2.5, 5.0, and 10.0 nm were obtained by simply changing the concentration of DA. The thickness of PDA and the carbonization temperature were crucial for the formation of porous structures. After carbonization at 900 °C, the 5.0 nm thick PDA/GO showed a rough surface and irregular mesopores of 3–5 nm, whereas the other samples only possessed random crinkles without a pronounced porous structure. For effective carbon precursor coating, Feng and coworkers used the amino-functionalized GO prepared by reacting the carboxylic acid groups of GO with 1,3-diaminopropane under the catalysis of N-hydroxysuccinimide and N-(3-(dimethylamino)propyl)-N′-ethylcarbodiimide hydrochloride [81]. It was demonstrated that the amino-functionalized GO effectively induced the growth of Schiff-base-type melamine-dialdehyde polymers [81] and boronic acid-functionalized polyaniline [84], which respectively resulted in nitrogen-doped(N-doped) mC/G and boron/nitrogen co-doped (B/N-co-doped) mC/G nanosheets after carbonization (Figure 4A). Instead of the growth of carbon precursors on GO, preformed carbon particles can be directly deposited on G without subsequent high temperature carbonization. The Fan group synthesized carbon black (CB)/G nanosheet (9:1 weight ratio) by the ultrasonication and in situ reduction methods. It was found that CB particles preferentially deposited on the edge surfaces of G by the ultrasonication method, and on the basal surfaces of G by in situ reduction method [99].

On the other hand, Zhong et al. proposed a zeolitic imidazolate framework (ZIF-8)-derived synthesis pathway to obtain N-doped mC/G [79]. Before ZIF-8 deposition, the surface of GO was functionalized with poly(vinyl pyrrolidone) (PVP). The amide carbonyl groups of PVP can coordinate with Zn ions and ensure the homogeneous nucleation of ZIF-8 on GO. The resulting ZIF-8/GO was carbonized at 800 °C for 5 h and washed with HCl to remove Zn species completely. On the other hand, Sun et al. developed a fabrication method for the growth of an ultrathin N-doped holey carbon layer on GO using the hybridized Zn gluconate as a carbon source and a porogen [80]. An aqueous mixture of Zn gluconate, ammonia, and GO was hydrothermally reacted at 180 °C for 3 h. The as-made hybrid was freeze-dried and grinded with urea (N source) before pyrolysis at various temperatures (600–1000 °C) under Ar. Zn species were carbothermally reduced and evaporated upon pyrolysis, leaving irregular random mesopores ~2 nm with a moderate surface area of 256 m^2^ g^−1^ (Figure 4B–D).

#### 2.3.2. Mesoporous Metal Oxide and Others/Graphene

Several synthetic methods have been reported to ensure the selective nucleation and growth of inorganic NPs (TiO_2_, SnO_2_, Sn, NiO, Co_3_O_4_ and FeWO_4_) on the surface of GO. Since the rates of hydrolysis and condensation of metal precursors are much higher than those of silicate or carbon precursors, a careful choice of reagents, solvents and pH is necessary.

Typical GO has surface functional groups such as carboxyl, hydroxyl and phenol groups which can serve as the anchoring sites for preformed inorganic NPs and the nucleation sites for in situ growth of inorganic NPs. In 2009, Honma’s group synthesized SnO_2_ NPs decorated G nanosheets. SnO_2_ NPs (~5 nm) were prepared by controlled hydrolysis of SnCl_4_ by NaOH and then mixed with rGO dispersion in ethylene glycol [85]. G nanosheets were homogeneously distributed between loosely packed SnO_2_ NPs, creating nanoporous structures with many void sites. In addition, direct growth of inorganics on functional groups of GO has frequently been reported. Luo et al. demonstrated the fabrication of SnO_2_/G through a controlled hydrolysis of tin salts (SnCl_2_∙2H_2_O) in a GO containing ethylene glycol-water solution at 120 °C under ambient pressure [86]. The SnO_2_/C was heat treated at 500 °C under Ar atmosphere to increase the crystallinity. The resulting SnO_2_/C was used as precursor to prepare SnS_2_/C via H_2_S annealing [86] or G-confined Sn nanosheets (G/Sn/G) via additional glucose coating and carbonization [87]. Similarly, TiO_2_@SnO_2_@G was synthesized by sequential growth of SnO_2_ and TiO_2_ NPs in acidic aqueous dispersion of GO [88]. MO/G has also been synthesized by in situ hydrothermal growth of MO NPs on GO and subsequent reduction/calcination [122,123]. Rooney and coworkers combined hydrothermal treatment with a water-in-oil emulsion system [93] to synthesize TiO_2_ QDs (6–8 nm)/G nanosheets. As the basal plane of GO was partially hydrophobic while the functional groups attached on the surface were hydrophilic, the GO sheets could restack around water droplets using CTAB as emulsifier. During hydrothermal treatment of these microemulsions, TiO_2_ was crystallized and GO was reduced to G, respectively.

Generally, the synthesized MO/G are prone to disintegrate or randomly aggregate themselves due to the favorable MO–MO interactions. To circumvent this problem, Yue et al. suggested double protection strategy in which additional G layers encapsulate the preformed Co_3_O_4_/G and NiO/G [94]. The pH of Co_3_O_4_/G solution and GO solution was controlled to pH 7–8 and pH 5–6, respectively. Addition of positively charged Co_3_O_4_/G to negatively charged GO solution led to coagulation of GO and Co_3_O_4_/G. Following hydrazine reduction produced G/MO/G/MO/G sandwich structures, where the MO particles were protected by the upper and lower G layers.

**Figure 5:**
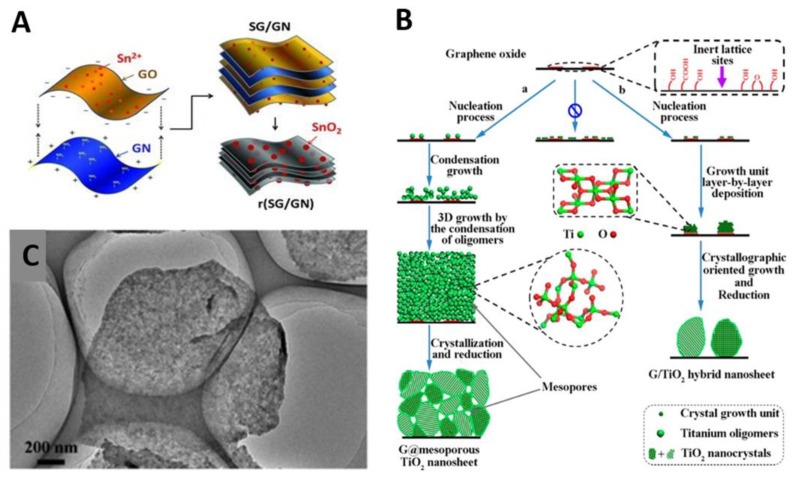
(**A**) Scheme of the fabrication of SnO_2_/G composites with alternating stacks of SnO_2_/GO and GN. Reprinted with permission from Ref. [90], Copyright (2013) John Wiley and Sons. (**B**) Scheme of the comparison of the amorphous-to-crystalline strategy for uniform mTiO_2_ deposition on G and conventional synthetic methodologies to growing TiO_2_ nanocrystals on G into hybrids. (**C**) TEM image of the TiO_2_/GO nanosheets with ammonia content of 0.15 mL. (**B**,**C**) Reprinted with permission from Ref. [97], Copyright (2015) American Chemical Society.

Although conventional GO itself has various surface functional groups, its density is usually low and sometimes insufficient to stabilize a large amount of MOs. Thus, undesired free growth of MOs in solution cannot be avoided in many cases. To solve these problems, researchers modified the surface chemistry of GO through heteroatom doping and surface functionalization. For example, N-containing functional groups can strongly anchor MOs to maintain the size of MO NPs [124]. Zhou et al. produced SnO_2_/N-doped rGO with pores (~2.2 nm) and nitrogen content (3 at%) via in situ hydrazine monohydrate vapor reduction method [89]. Hydrothermally synthesized SnO_2_ NPs (4–5 nm) were mixed with aqueous GO suspension and freeze-dried to form SnO_2_/GO composites. The obtained hybrid was reduced by hydrazine monohydrate vapor at 120 °C for 2 h, yielding SnO_2_/N-doped rGO (N-rGO) with exceptionally high SnO_2_ loading (70 wt%). SnO_2_ NPs were homogeneously distributed onto the N-rGO and their sizes remained unchanged when applied as anodes for LIBs. This result is mainly attributed to the formation of Sn-N bonds between SnO_2_ and G which effectively stabilize the SnO_2_ NPs on N-rGO. In addition, Lu and coworkers prepared N-rGO through thermal reduction of GO with urea, and subsequently used the N-rGO as a substrate that directed the growth of atomically thin mesoporous Co_3_O_4_ layers [98]. Pyo and coworkers employed an amine-functionalized graphene (GN) to fabricate SnO_2_/G composites with alternating stacks of SnO_2_/GO and GN [90]. The opposite surface charge of SnO_2_/GO (negative) and GN (positive) at pH 2 led to spontaneous formation of alternating stacks in aqueous solution (Figure 5A). Likewise, Feng’s group presented direct synthesis of 2D SnO_2_/G nanosheets through electrostatic interaction between in situ hydrolyzed Sn salt (negative) and PDDA-functionalized GO (positive) [91]. Colberg’s group used 7,7,8,8-tetracyanoquinodimethane anion (TCNQ^−^) as both the nitrogen source and complexing agent to develop sandwich-like SnO_2_/N-rGO [92]. The addition of Sn (II) salt to the TCNQ/G solution induced self-assembly into the sandwich structures due to the strong electrostatic interaction between Sn^2+^ and TCNQ^-^. The use of additional linker-molecules that increase the interaction between GO and inorganics is also suggested. Qui et al. used glucose as the linker and the face-controlling agent for hydrothermal growth of mesoporous TiO_2_ NPs on a G aerogels [95]. Because of many hydroxyl groups on glucose, glucose molecules preferably adsorbed onto the (001) surfaces of TiO_2_, forming TiO_2_ seeds with exposed (001) facets. Hydroxyl groups also served as the linker, allowing the in situ growth TiO_2_ on GO.

On the other hand, Zhao and coworkers proposed a sol-gel design strategy for ultradispersed TiO_2_ NPs on G [96], without employing additional functionalization steps or linker molecules. A pure ethanol system with an ultralow content of ammonia (0.1 v%) was used to slow the hydrolysis and condensation of titanium alkoxides. The mixture of ethanol, ammonia and a titanium source was stirred at 25 °C for 24 h. Amorphous TiO_2_ NPs (~5 nm) were deposited at the oxygen functional group sites in the GO domain owing to their strong interactions with titanium oligomers in a covalent manner. However, TiO_2_ only showed discontinuous island-like morphologies due to the limited compatibility between TiO_2_ and GO. Thus, the same group further modified this strategy to achieve continuous, conformal coating of amorphous TiO_2_ shells on GO sheets [97]. The dispersion, density, and thickness (10~45 nm) of TiO_2_ layers were controlled by tuning the content of ammonia (0.10~0.45 v%). Ammonia greatly retarded hydrolysis and condensation of titanium alkoxides and thus facilitated heterogeneous nucleation of TiO_2_ on GO surface. Then, the newly hydrolyzed Ti-oligomers slowly diffused and polymerized around the TiO_2_ nuclei, forming a 3D cross-linked and continuous TiO_2_ layers (Figure 5B,C). Subsequent thermal annealing at 500 °C converted amorphous TiO_2_ layers into crystalline mesoporous TiO_2_ shells encapsulating the G sheets.

### 2.4. Top-Down Method

Preformed G or GO can be nano-engineered to obtain 2D porous G materials, which has been realized by chemical etching or activation generating nanopores without specific templates. The chemical etching involves etching agent such as base (e.g., KOH) [100,101,102], acid (e.g., HNO_3_) [103,104,125,126], metal NPs (e.g., Ag and Au) [105,127] and MO NPs (e.g., KMnO_4_, Co_3_O_4_, and Fe_2_O_3_) [106,107,108,109,128], H_2_O_2_ [110,129,130] and gases (e.g., steam and air) [111,112,113].

#### 2.4.1. KOH Activation

KOH is a common activation agent that allows the production of porous activated carbon with high specific surface area. The KOH activation process involves the decomposition of C atoms on carbon surfaces via several reactions as follows [131]:

6KOH + 2C → 2K_2_CO_3_ + H_2_ + 2K

K_2_CO_3_ → K_2_O → CO_2_

K_2_CO_3_ + C → K_2_O → 2CO

K_2_O + C → 2K + CO

**Figure 6:**
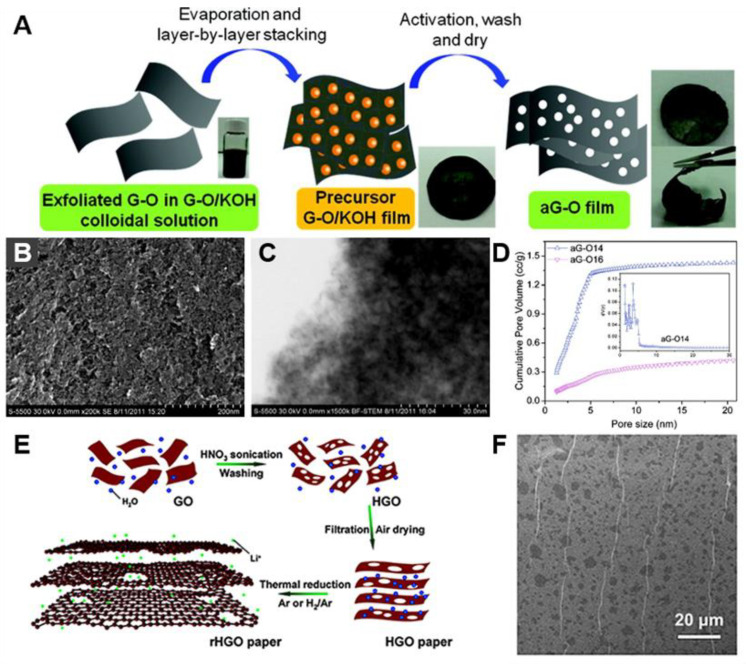
(**A**) Scheme of the preparation of microwave exfoliation of GO (MEGO) and the following activation process with KOH to generate nanopores. (**B**) High-resolution scanning electron microscopy (SEM) image, (**C**) Gas (N_2_, Ar, and CO_2_) physisorption isotherm of an a-MEGO, and (**D**) cumulative pore volume and pore size distribution (inset). (**A**–**D**) Reprinted with permission from Ref. [102], Copyright (2012) American Chemical Society. (**E**) Scheme of the formation of in-plane pores into GO by HNO_3_ etching and (**F**) SEM image of holey GO sheets. (**E**,**F**) Reprinted with permission from Ref. [103], Copyright (2011) American Chemical Society.

The KOH activation was employed to produce in-plane pores in G [100,101,102]. The KOH activation of G underwent the aforementioned processes in which C atoms were removed and oxidized into CO or CO_2_ to generate pores. For example, 2D nanoporous G was synthesized by the KOH activation method of rGO (Figure 6A) [102]. The porous microwave exfoliated GO (MEGO) had well-defined micro- and mesopores in the range from 0.6 to 5 nm and a high surface area of 3100 m^2^ g^−1^ (Figure 6B–D). KOH-activated porous GO also showed improved electrical conductivity of ~500 S m^‒1^ due to low H content and a high C/O atomic ratio of 35 [100]. The mass ratio of KOH to GO and the activation temperature greatly affected the properties of porous MEGO (e.g., surface area, pore volume, and specific capacitance) by tuning the degree of activation [101,102]. With an increase of KOH/GO ratio from 0 to 6.5, Brunauer-Emmett-Teller (BET) surface area increased from 304 to 3100 m^2^ g^−1^. However, further increase of KOH/GO ratio resulted in decrease of the BET surface area. It was also found that porous MEGO activated at 800 °C had the highest surface area of 3100 m^2^ g^−1^ [101].

#### 2.4.2. Acid Etching

Acid etching is another effective approach to produce porous G. The combination of the concentrated HNO_3_ and ultrasonic wave can cut GO nanosheets into polyaromatic carbon species because of high strain and frictional forces [132]. Therefore, an acid etching method that integrates mild HNO_3_ condition and sonication was introduced to generate in-plane pores in GO nanosheets (Figure 6E) [103]. HNO_3_ can react with the unsaturated C atoms at defect sites and edge sites of GO nanosheets. The HNO_3_ etching partially removed and detached C atoms of G, resulting in holey GO nanosheets (Figure 6F). The etching process dramatically increased the amount of the epoxy/hydroxyl (C‒O) group and the carbonyl (C=O) group, while reducing the amount of aromatic C‒C bonds. The pore size was controlled from 7 to 600 nm by changing the HNO_3_ concentration. The solution-based acid etching also can be used for the large scale preparation of nanoporous graphene nanomeshes (GNMs) [104]. The rGO sheets were refluxed in concentrated HNO_3_ solution at 100 °C, resulting in GNMs with disordered porous structures and irregular nanopores. The porosity and the pore size of GNMs were easily tunable by varying the time of acid treatments. The number density of nanopores in GNMs decreased from 55 to 15 μm^−2^, but pore size increased from 10 nm to hundreds of nanometers with the increase of the HNO_3_ reflux time from 4 to 9 h. After the reflux time of 11 h, the GNMs were broken into small nanosheets.

#### 2.4.3. Catalytic Oxidation

**Figure 7:**
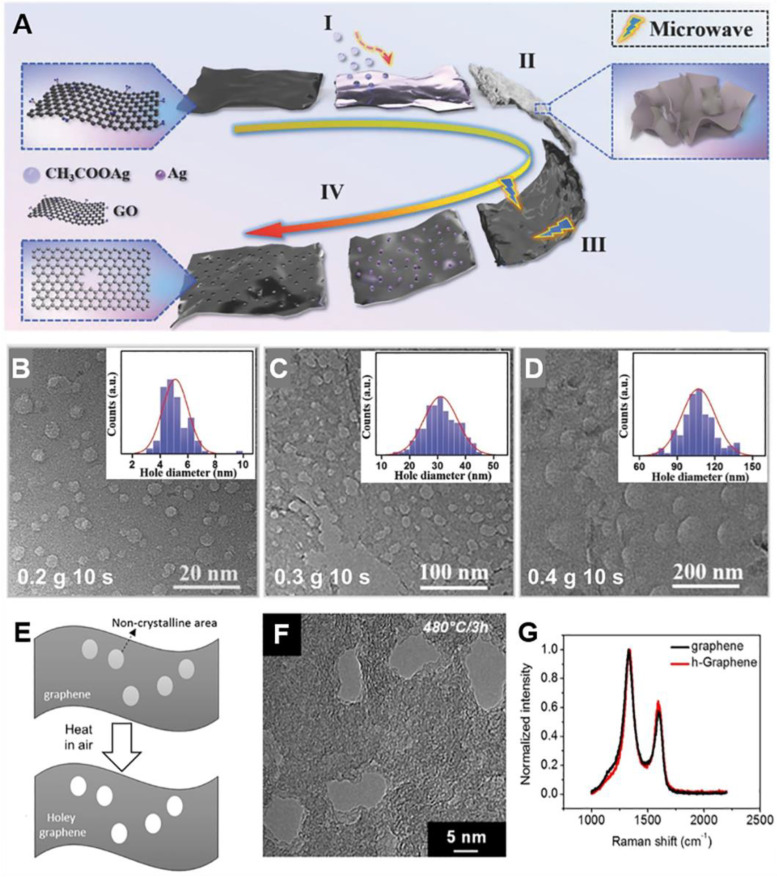
(**A**) Schematic of the fabrication for porous graphene. (**B**–**D**) HRTEM images of various porous graphene prepared by different concentrations of Ag acetate after microwave treatment for 10 s and corresponding pore diameter distribution (inset). (**A**–**D**) Reprinted with permission from Ref. [105], Copyright (2018) John Wiley and Sons. (**E**) Schematic of the synthesis, (**F**) TEM image, and (**G**) Raman spectra comparison of holey graphene prepared by an air-etching approach. (**E**–**G**) Reprinted with permission from Ref. [112], Copyright (2014) American Chemical Society.

Catalytic oxidation methods with metal NPs, MO NPs, or metal compounds have been commonly used for the chemical etching of G to prepare porous G. These approaches are based on the redox reaction mechanism: the oxidation of carbon in G and the reduction of metal compounds. For example, porous rGO nanosheets were prepared by using Ag NPs under microwave combustion process [105]. Oxygen molecules adsorbed on the surface of Ag NPs first diffused into the reaction site. Then Ag NPs led to the selective oxidation of carbon in contact with NPs into CO or CO_2_ under microwave treatment. Subsequent removal of metal NPs by an acid treatment yielded porous rGO nanosheets with uniform nanopores and a specific surface area of up to 965 m^2^ g^−1^. This method could be utilized with other metal precursors (e.g., CH_3_COOAg, Co(NO_3_)_2_, and Cu(NO_3_)_2_). The metal precursors were reduced to metal NPs by microwave irradiation, which catalytically improved the carbon oxidation around NPs (Figure 7A). Catalytic oxidation methods also could readily control the pore size by changing the NP size or the amount of metal precursors. This was due to the fact that the pore size corresponds to the size of NP which contacts with rGO nanosheets. The different pore sizes of 5, 30, and 100 nm were achieved by changing the amounts of Ag acetate in GO solution (Figure 7B–D). The larger amount of precursor formed bigger metal NPs during the microwave combustion process, which increased the generated pore size. KMnO_4_ was also used as a catalyst to produce porous rGO nanosheets by reducing aqueous MnO_4_^−^ into MnO_2_ under microwave treatment, resulting in the specific surface area of 1374 m^2^ g^−1^ and mesopores of 2.4 nm [106].

The carbothermal reduction between G and metal oxide NPs was also utilized to synthesize 2D porous G [107,108,109]. Ren et al. reported mesoporous G nanosheets with uniform and tunable pores prepared by the carbothermal reduction of Co_3_O_4_ NPs [107]. During heat treatment at 900 °C under Ar, Co_3_O_4_ reduced to Co and CoO with consumption of C atoms in GO nanosheets. Co_3_O_4_ NPs generated uniform mesopores with an average pore size of 21 and 53 nm, corresponding to Co_3_O_4_ NP size. The etching of GO with Fe_2_O_3_ NPs derived from ferrocene was the solution-free carbothermal reduction method for the synthesis of holey G nanosheets [108]. Ammonium-containing polyoxometalate (POM) was employed to directly prepare N-doped porous G [109]. Oxometalates (OMs) and POMs also reduced to metal, metal oxides or metal carbides during the carbothermal reduction at 650 °C under N_2_ atmosphere, leading to porous G nanosheets with mesopores of 20–50 nm. During thermal decomposition, NH_3_-containing (NH_4_)_6_Mo_7_O_24_ generated gaseous NH_3_ which is the N-doping agent of mesoporous N-doped G. Their N content was 4.54 at%, which is comparable to the NH_3_-assisted N-doping method.

#### 2.4.4. Other Oxidative Agents

H_2_O_2_ and some gases (e.g., steam and air) as oxidants also partially oxidized the C atoms around the defect sites and edges in the basal plane of G, creating various nanopores. H_2_O_2_ oxidatively etched and removed the oxygenated C atoms and generated carbon vacancies by thermal treatment of an aqueous solution of GO and H_2_O_2_ at 100 °C [110]. Further reaction between GO and H_2_O_2_ gradually extended carbon vacancies to in-plane nanopores. The pore size enlarged with the increase of reaction time and the H_2_O_2_ concentration from 2 nm to hundreds of nanometers [129]. Han et al. showed a controllable steam etching method to produce nanoporous rGO sheets by hydrothermal steaming at 200 °C [111]. H_2_O vapor reacted with C atoms, and defect sites of GO were decomposed into H_2_ and CO, which yielded carbon vacancies. The steam etched porous G shows highly developed nanoporous networks extended from small nanopores and cracks. The slow etching process of the steam method allowed sufficient time to control the porosity. An air-etching approach was also demonstrated as a scalable one-step synthesis of holey G sheets with a chemical-free procedure [112]. Oxygen selectively reacted with the non-crystalline region of the basal plane of G in the air at 480 °C, leaving pores by decomposing to CO_2_ (Figure 7E). The produced holey G has the improved surface area (658 m^2^ g^−1^) and narrowly distributed mesopores (Figure 7F). After heating at 480 °C for 3 h in air, the D-to-G ratio in Raman spectroscopy slightly increased due to moderate heating condition (Figure 7G). In the reaction temperature range (430–480 °C), sp^2^ carbons in the graphitic region remained stable, while sp^3^ carbons in the defective region are readily removable by oxidation. The size, morphology, and density of pores were tunable by the reaction condition of heating rate, temperature, and reaction time. Further heating of holey G under Ar atmosphere at 900 °C affected pore size, pore volume, and surface area [113].

## 3. Energy Storage Applications

Based on the synthesis strategies described in Section 2, a wide variety of G-based 2D mesoporous materials have been developed as promising electrode materials in SCs (Table 2) and LIBs (Table 3). In both devices, the as-made GO-hybrid materials must undergo thermal or electrochemical reduction processes to restore the beneficial properties of G such as high thermal/electrical conductivity. The degree of graphitization and the amount of surface functional groups and impurities should be carefully controlled as they have a great influence on the electrochemical performance of the final materials. Since the required material properties greatly depend on the energy storage mechanism, the physical properties of G-based 2D mesoporous materials such as pore size, structures as well as particle size and morphology should be tailored to the target applications. For instance, a high specific surface area is highly advantageous for the surface-driven charge storages in SCs, whereas it can promote the undesired irreversible Li^+^ ion consumption in LIBs. For SCc, mesoporous materials with high surface area and hierarchical micro-meso-macroporous structures (e.g., carbon and pseudocapacitive metal oxides) are highly desirable to increase the energy and power density of electrodes. For LIBs, mesoporous materials with low surface area and high intrinsic capacity (e.g., Sn, Si) are attractive candidates. The intimate contact between high capacity mesoporous materials and G is necessary to maintain continuous electron-conductive path. In addition, the pore size and structures are important to alleviate the stress from huge volume changes during repeated cycling. The mesopores and macropores can enhance the rate capability of LIBs especially at high C-rates. In the following sections, we introduced the representative G-based 2D mesoporous materials used in SCs (Section 3.1) and LIBs (Section 3.2) and highlighted the strategies used to improve the electrochemical performance in each device.

### 3.1. Supercapacitors

SCs have attracted great attention because of their fast charge/discharge rates, high power densities, and excellent cycling stability, so they have been used in various electronic devices [40,137,138]. SCs can be classified into two types depending on the energy storage mechanism: the electrical double-layer capacitors (EDLCs) and pseudocapacitors (PCs). EDLCs involve electrostatic adsorption of electrolyte ions on the surface of the electrode materials during the charge and discharge processes, not electrochemical reactions. PCs store and release charges by the surface faradaic redox reactions of electrode materials. The energy storage of SCs originates from the reversible reaction on the surface of electrode materials, including charge separation and faradaic reaction. Their charge storage mechanism realizes remarkable power densities and rate capability, but relatively low energy densities compared with rechargeable batteries (e.g., LIBs). Recently, hybrid supercapacitors (HSCs) with an asymmetric electrode configuration have been extensively studied to improve energy density [138,139,140,141,142]. The hybrid system integrates the SC-type cathode with the battery-type anode to simultaneously achieve high energy and power density with excellent cycling stability.

**Figure 8:**
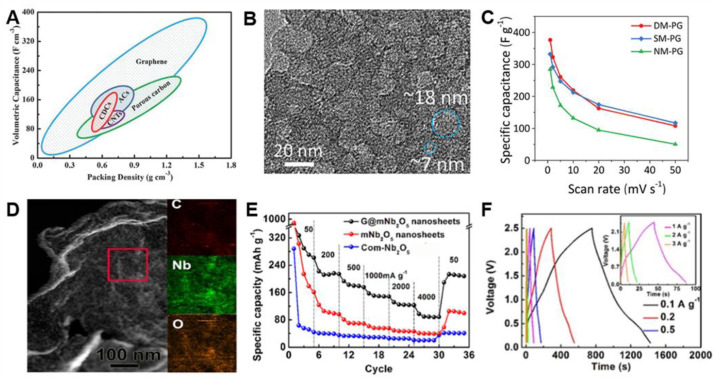
(**A**) Volumetric capacitance and packing density of different carbon electrodes. Reprinted with permission from Ref. [22], Copyright (2018) John Wiley and Sons. (**B**) TEM image of DM-PG nanosheets with dual mesopores of 7 and 18 nm. (**C**) Specific capacitance as a function of scan rate. (**B**–**C**) Reprinted with permission from Ref. [133], Copyright (2020) John Wiley and Sons. (**D**) STEM image of G@mNb_2_O_5_ nanosheets and the corresponding energy-dispersive X-ray spectroscopy (EDX) analysis of C, Nb, and O. (**E**) Rate performance of G@mNb_2_O_5_, mNb_2_O_5_ nanosheets and commercial-Nb_2_O_5_ electrodes. (**F**) Charge/discharge performance of G@mNb_2_O_5_//AC hybrid supercapacitor at various current densities from 0.1 to 3 A g^−1^. (**D**–**F**) Reprinted with permission from Ref. [136], Copyright (2017) Elsevier.

G-based 2D materials are attractive SC electrode materials due to high surface area, excellent electrical conductivity, high packing density, and high mechanical/chemical stability (Figure 8A) [22]. Furthermore, the 2D dimensional structures allow the fabrication of high-density electrodes with high volumetric capacitance. Therefore, G-based materials have been considered ideal electrode materials for high performance SCs. However, the self-restacking of G sheets significantly reduces accessible surface area and the ion transport pathway, resulting in poor device performance. The introduction of the porous structure can overcome the intrinsic disadvantages of G-based materials to improve the electrical performance of SCs. The porous structure can prevent restacking of G sheets, provide numerous active sites, and accelerate their ion transport. Various strategies have been employed, such as the use of porous materials as a spacer on G sheets, 3D structure engineering, and generation of in-plane pore on G sheets, which are described in more detail in the following.

Mesoporous material (e.g., carbon and metal oxides) on the G sheets can be used as a spacer, which hampers restacking G sheets and provides additional capacitance [74,82,133,136,143,144,145,146,147]. Mesoporous carbon/G nanocomposite (mCG) synthesized by KIT-6 had a surface area of 1179 m^2^ g^−1^ and bimodal pores of 3.1 and 3.7 nm [74]. The specific capacitance of mCG was up to 276 F g^−1^ at the current density of 1 A g^−1^. mCG exhibited 97% of capacitance retention with the current density increase from 1 to 20 A g^−1^. Furthermore, it maintained 86% of the initial capacitance at 100 A g^−1^. Mesoporous carbon (mC) showed a comparable specific capacitance (247 F g^−1^) at 1 A g^−1^, but its capacitance significantly decreased to 61 F g^−1^ at 20 A g^−1^, indicating low capacitance retention of 25%. The enhanced rate capability of mCG over mC was attributed to the high electrical conductivity from G and improved ion transport within 2D mC on G. On the other hand, incorporation of CB particles (20–50 nm) into G layers can improve not only electrolyte-electrode accessibility but also electrode conductivity, because CB particles as the spacer can provide a rapid diffusion path in double-layer capacitance. CB/G composites showed the high specific capacitance of 118.1 F g^−1^ even at 500 mV s^−1^, which is higher than that of pure G nanosheets [99].

Recently, 2D sandwich-like mesoporous polypyrrole (PPy)/G (PG) was prepared by using dual templates (SiO_2_ sphere and PEO-*b*-PS) for generation of bimodal mesopore (7 and 18 nm) (Figure 8B) [133]. PPy has high electron affinity, reversible redox activity, and tunable electrical conductivity, so it has great potential as a promising high-pseudocapacitive material for SCs. The combined properties of pseudocapacitive PPy and conductive G synergistically improved the electrochemical performance of SC. Dual-mesoporous PG (DM-PG) exhibited a higher specific capacitance of 376 F g^−1^ at 1 mV s^−1^ than single-mesoporous PG (SM-PG) (332 F g^−1^) and non-mesoporous PG (NM-PG) (284 F g^−1^). Moreover, DM-PG and SM-PG delivered a high-rate capability of 108 and 117 F g^−1^ at a high scan rate of 50 mV s^−1^, respectively, which is higher than that of NM-PG (51 F g^−1^) (Figure 8C). Both DM-PG and SM-PG maintained 94% of initial capacitance after 3000 cycles (60% for NM-PG), indicating that mesopores also allow PG nanosheets to improve cyclability in SCs.

Mesoporous metal oxides (e.g., WO_3_, Nb_2_O_5_, TiO_2_) and G composite can be utilized as an electrode in SC, especially Li- or Na-ion HSCs [136,144,146]. The sandwich-structured mesoporous Nb_2_O_5_/G/mesoporous Nb_2_O_5_ (G@mNb_2_O_5_) was used as an anode material in Na-ion HSCs (Figure 8D) [136]. G@mNb_2_O_5_ nanosheets possessed broad mesopores from 1 to 4 nm and the specific surface area of 366 m^2^ g^−1^, which is higher than that of mesoporous Nb_2_O_5_/carbon composite (96 m^2^ g^−1^) and Nb_2_O_5_ NP/rGO composite (253 m^2^ g^−1^). Nano-sized mesoporous Nb_2_O_5_ layers provided a short Na-ion pathway for fast diffusion, the surface pseudocapacitive reaction, and improved Na-ion intercalation. Therefore, G@mNb_2_O_5_ exhibited the specific capacity of 293 mA h g^−1^ at 50 mA g^−1^ and a high-rate capability 125 mA h g^−1^ at 2000 mA g^−1^ with stable cyclability over 2000 cycles (Figure 8E). By employing G@mNb_2_O_5_ as an anode and activated carbon (the specific surface area of 2084 m^2^ g^−1^) as a cathode, the Na-HSC showed a high energy density of 56.1 W h kg^−1^ at a power density of 120 W g^−1^ (Figure 8F) and stable capacity retention of 89% after 800 cycles at 1 A g^−1^.

**Figure 9:**
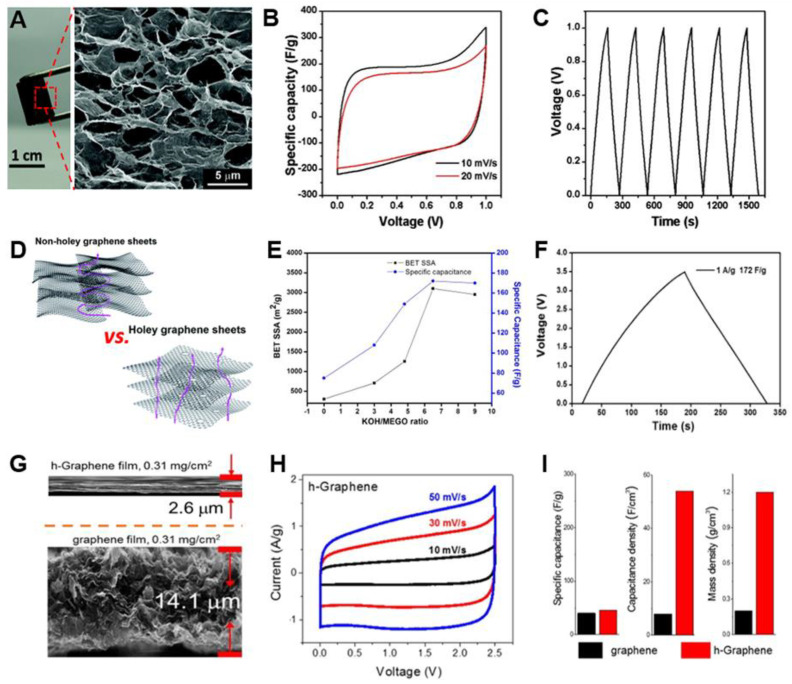
(**A**) Photograph and SEM images of the SGH material. (**B**) Cyclic voltammograms of the SGH-based supercapacitor at two different scan rates. (**C**) Galvanostatic charge/discharge curves of the SGH-based supercapacitor at a constant current of 1 A g^−1^. (**A**–**C**) Reprinted with permission from Ref. [134], Copyright (2010) American Chemical Society. (**D**) Schematic representation of the comparison of the ion transport pathway of holey G sheets and non-holey G sheets. Reprinted with permission from Ref. [150], Copyright (2020) The Royal Society of Chemistry. (**E**) Effect of activation KOH/MEGO ratio on the surface area and specific capacitance of a-MEGO. (**F**) Galvanostatic charge/discharge curves of a-MEGO-based supercapacitor at different current densities. (**E**,**F**) Reprinted with permission from Ref. [101], Copyright (2012) Elsevier. (**G**) Cross-sectional SEM images of h-G and G films. (**H**) Cyclic voltammogram of the h-G electrode at various voltage scan rates. (**I**) Comparison of gravimetric and volumetric capacitance. (**G**–**I**) Reprinted with permission from Ref. [112], Copyright (2014) American Chemical Society.

3D structure engineering by self-assembly of G sheets is an effective strategy to prevent their restacking problems [134,135,148,149]. 3D macroscopic G architecture provides fast ion diffusion through macropores, high mechanical strength, and outstanding conductivity properties. The macroporous structure also ensures a sufficient wettability of the electrode-electrolyte interface. These properties lead to an increase in the rate capability of SCs. Shi and co-workers reported the direct self-assembly approach of G into 3D structured G frameworks with macropores by a hydrothermal method (Figure 9A) [134]. The self-assembled G hydrogel (SGH) had high electrical conductivity (5 × 10^−3^ S cm^−1^) and a higher modulus of 490 kPa than that of the conventional hydrogels. The specific capacitances of SGH were 175 and 152 F g^−1^ at 10 and 20 mV s^−1^, respectively, in 5M KOH electrolyte (Figure 9B,C). This result was 50% higher than that of rGO agglomerated particle electrode (100 F g^−1^ at 20 mV s^−1^). Macroporous nitrogen-doped G hydrogel (GN-GH) was also prepared by the hydrothermal method with an organic amine [135]. The GN-GH electrode exhibited remarkably enhanced SC performance, such as a high specific capacitance of 190 F g^−1^ and a power density of 245 kW kg^−1^ at 10 A g^−1^. Even at the ultrafast current density of 250 A g^−1^, the specific capacitance of 109.4 F g^−1^ and the power density of 173 kW kg^−1^ were achieved. Compared with G hydrogel (G-GH), its specific capacitance and power densities were 69 F g^−1^ and 38 kW kg^−1^ at 100 A g^−1^, which are much lower than those of GN-GH.

Holey G sheets with in-plane nanopores (e.g., micropore, mesopore, or both) have beneficial properties for SCs as electrodes compared with non-holey G sheets: short ion diffusion pathway to a vertical direction and a large number of active sites at the holey edges (Figure 9D) [150]. Ruoff and co-workers reported micro- and mesoporous activated microwave exfoliated GO (a-MEGO) by using KOH solution [100]. The surface area of a-MEGO was 3100 m^2^ g^−1^ and the micro- and mesopores were distributed in the range of 1–10 nm. The specific surface area of a-MEGO can be controlled from 760 to 3100 m^2^ g^−1^ by varying the carbonization temperature and the ratio of KOH/MEGO (Figure 9E) [101]. With the organic electrolyte, a-MEGO shows 172, 165, 166, and 166 F g^−1^ at current densities of 1, 1.4, 2.8, and 5.7 A g^−1^, respectively (Figure 9F) [101,102]. The energy density was calculated to ~70 W h kg^−1^ at the working voltage of 3.5 V, which is much higher than that of activated carbon-based SCs (20 W h kg^−1^). Furthermore, the a-MEGO showed superior cyclability; 97% of initial capacitance was maintained after 10,000 cycles at 2.5 A g^−1^. Holey G sheets (h-G) also remarkably improved volumetric capacity because of their closely packing properties with excellent ion transport [112,129]. The h-G with mesopores generated by the air-etching approach showed the increased surface area of 658 m^2^ g^−1^ compared with the starting G (471 m^2^ g^−1^) [112]. When h-G and G films were fabricated by filtration method, the thickness of the h-G film (2.6 μm) was thinner than that of G film (14.1 μm), indicating the density of h-G film (1.2 g cm^−3^) is 6 times higher than G film (0.2 g cm^−3^) (Figure 9G). It is because unreleased solvent occupies between G sheets, resulting in void space after solvent evaporation. In contrast, the residual solvent quickly escaped from h-G through mesopores. This property resulted in high volumetric capacitance of the h-G electrode, which delivers 54 F cm^−3^ at a current density of 3 A g^−1^ (Figure 9H,I). The G electrode showed a low volumetric capacitance of 8 F cm^−3^, only 15% of the h-G electrode. The h-G electrode also exhibited excellent cycling stability at the current density of 3 A g^−1^. The capacity retention was 53 F cm^−3^ after 10,000 cycles and it retained 98% of the initial capacitance with a high coulombic efficiency (CE) of 99%. The volumetric energy density of h-G was 12 W h L^−1^ at 3 A g^−1^, which is higher than that of the pristine G (~2 W h L^−1^).

### 3.2. Lithium-Ion Batteries

**Figure 10:**
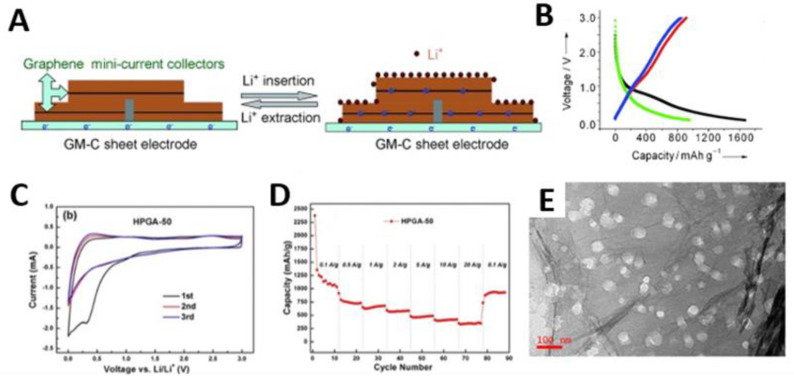
(**A**) Lithium insertion and extraction in mC/G sheets, where G acts as mini-current collectors during discharge and charge processes. (**B**) First two discharge-charge curves (first discharge (black), first charge (red), second discharge (green), second charge (blue)). (**A**,**B**) Reprinted with permission from Ref. [55], Copyright (2010) John Wiley and Sons. (**C**) First three cyclic voltammograms of hierarchical porous graphene aerogels (HPGA) anode at a scan rate of 1 mV s^−1^ between 0.1–3.0 V. (**D**) Rate capability of the HPGA-50 anode. (**E**) TEM image of HPGA-50. (**C**–**E**) Licensed under CC-BY from Ref. [107].

In LIB, graphite is the most commercially available anode material due to its good electrical conductivity and high chemical stability. However, low theoretical capacity of graphite (372 mAh g^−1^) limits the development of LIB with high energy density [51]. As an alternative to conventional graphite, G-based 2D mesoporous carbons have been proposed as potential anode materials for LIB. As an example, the Müllen group employed mSiO_2_/G as a template with sucrose as carbon source to synthesize mC/G sheets and tested anode performance (Figure 10A,B) [55]. Galvanostatic charge/discharge profiles showed excellent Li^+^ ion storage properties with the reversible capacity of 370 mAh g^−1^ at 5 C. These results indicate that the advantageous 2D mesoporous structures can improve the electrochemical performance compared to conventional non-graphitic carbons [151] or porous graphitic carbons [152]. In addition, porous G nanosheets (PGN) have attracted enormous research interest. Their unique porous structure in combination with inherent properties of G such as large specific surface area and excellent conductivity can improve the electrochemical performance. The PGN electrodes exhibited excellent rate capability and a high reversible specific capacity of 963.4 mAh g^−1^ at 100 mA g^−1^ after 200 cycles. Among PGN, hierarchically porous G-based materials showed great potential as anodes for LIB because of the combined advantages of the different pore size regimes [153]. Macropores serve as electrolyte reservoirs, facilitating electrolyte wetting and mass transport of electrolytes. Mesopores further shorten the solid-state Li^+^ ion diffusion distances, accelerating Li^+^ ion diffusion kinetics. Micropores increase the specific surface area of the materials which would increase the number of electrochemically active reversible Li^+^ storage sites [24]. The Li^+^ ion storage properties of 3D hierarchical porous G aerogels (HPGA) with 2D mesoporous G nanosheets were evaluated (Figure 10C–E) [107]. The HPGA anode had not only a high reversible capacity of 1100 mAh g^−1^ at a current density of 0.1 A g^−1^, but also superior cycling stability and excellent rate performance. These excellent electrochemical properties were attributed to their unique structures which offer the 3D pore networks for facile Li^+^ ion adsorption and thin graphitic layers for Li^+^ ion intercalation.

**Figure 11:**
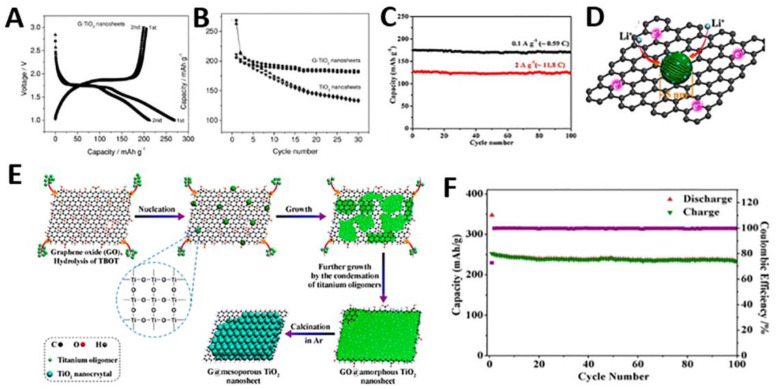
(**A**) First two charge-discharge curves of TiO_2_/G nanosheets at a current density of 0.2 C. (**B**) Cycling performance of TiO_2_/G and TiO_2_ nanosheets at a current density of 0.2 C. (**A**,**B**) Reprinted with permission from Ref. [75], Copyright (2011) John Wiley and Sons. (**C**) Cycling performance of the TiO_2_/rGO sheet electrode at constant density of 0.1 and 0.2 A g^−1^. (**D**) Schematic representation of the electrochemical reaction path on the TiO_2_ nanocrystals/rGO sheets. (**C**,**D**) Reprinted with permission from Ref. [96], Copyright (2013) American Chemical Society. (**E**) Schematic formation process of the mTiO_2_/G/mTiO_2_ sandwich-like nanosheets. (**F**) Plots of specific capacity and CE of the mTiO_2_/G sandwich-like nanosheet electrode as a function of cycle number at a constant current density of 20 mA g^−1^. (**E**,**F**) Reprinted with permission from Ref. [97], Copyright (2015) American Chemical Society.

Titanium-based anode materials have been popularly applied to LIB because of high lithium insertion/extraction voltage at about 1.7 V (*vs.* Li/Li^+^). This high insertion voltage greatly decreases the risk of formation of the solid electrolyte interphase (SEI) layers and lithium dendrites. Furthermore, TiO_2_ is an abundant, low-cost and environmentally benign material with a low volume change (3–4%), which is necessary for mass production of energy storage devices. However, the use of TiO_2_ for LIB applications has been largely restricted due to its low ionic and electrical conductivity, which is rate determining for the lithium insertion and extraction. Therefore, researchers have used various conductive additives to improve its electrochemical performance. In this respect, G would be an ideal conductive substrate to stabilize TiO_2_ crystals and increase electrical conductivity and rate capability. The Müllen group synthesized TiO_2_/G nanosheets and compared them with TiO_2_ nanosheets, highlighting the role of G layers within each TiO_2_ nanosheet (Figure 11A,B) [75]. As an anode material for LIB, TiO_2_/G nanosheets exhibited a high first discharge capacity of 269 mAh g^−1^ at a current rate of 0.2 C. Although this capacity was close to that of TiO_2_ nanosheets without G at the first cycle, the difference in capacity loss between TiO_2_ and TiO_2_/G increased during cycling. The G layer within each nanosheet can serve as a mini-current collector allowing fast electron transport in the electrode. TiO_2_/G nanosheets exhibited a higher lithium insertion coefficient with respect to pure TiO_2_, demonstrating the existence of addition lithium storage sites.

However, a weak interaction between TiO_2_ NPs and G surface could hinder the transfer of electrons and Li^+^ ions. Therefore, researchers have investigated various approaches for uniform oxide deposition on G sheets. To increase the interaction between GO and inorganic materials, glucose as the linker was used to enhance the dispersion of TiO_2_ nanocrystals on G aerogels surfaces [95]. Mesoporous TiO_2_ NPs on a G aerogels achieved high capacity, stable cycle performance and improved reversibility (~202 mAh g^−1^ at 0.59 C) due to the strong connection between TiO_2_ and G. Similarly, anionic sulfate surfactants were also used to increase interactions between G and oxide materials, resulting in improved specific capacity [68]. Mo et al. synthesized TiO_2_ QDs/G nanosheets through a water-in-oil emulsion system [93]. The specific capacity of this nanosheet was 101 mAh g^−1^ at the high rate of 50 C. The excellent electrochemical performance would be due to the well-dispersed TiO_2_ QDs which could avoid the aggregation of titania clusters and offer a direct pathway for Li^+^ diffusion.

The ultradispersed TiO_2_ NPs on G were obtained through a sol-gel design strategy (Figure 11C,D) [96]. These small TiO_2_ NPs (~5 nm) improved the theoretical lithium capacity (x > 0.5) for anatase Li_x_TiO_2_ and, therefore, the capacity reached up to 352 mAh g^−1^ in the first discharge at 0.59 C. Subsequently, the same group developed an amorphous-to-crystalline strategy for uniform mTiO_2_ deposition on G sheets (Figure 11E,F). They obtained thin mesoporous TiO_2_ layer, uniform mesopores (~3.4 nm) and high surface area (~252 m^2^ g^−1^) by coating G sheets with continuous TiO_2_ layers [97]. In this study, the maximized junction areas between G and TiO_2_ nanocrystals increased interfacial surface areas for lithium storage. Therefore, the resulting nanosheets showed a stable cyclability, high CE, and a reversible capacity of ~237 mAh g^−1^ at a constant current density of 20 mA g^−1^.

**Figure 12:**
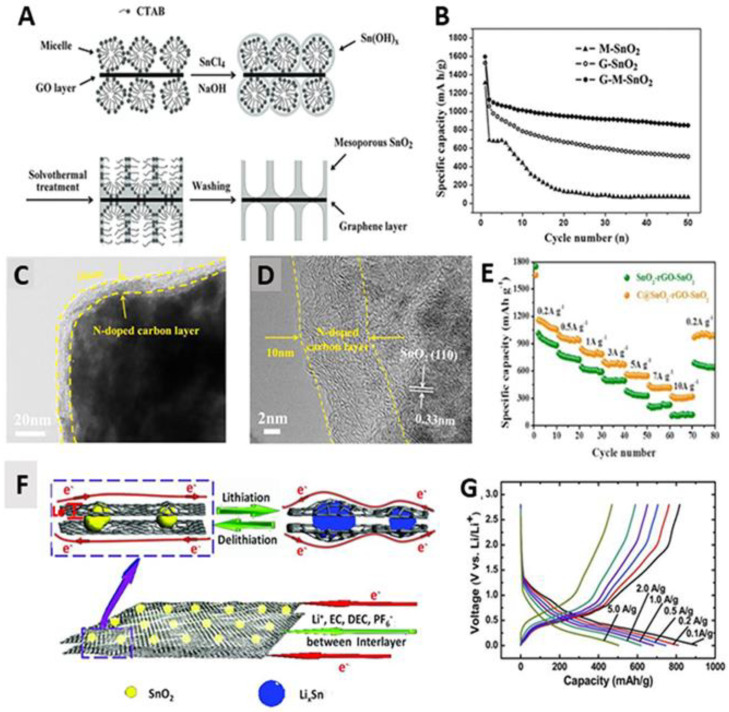
(**A**) Schematic mechanism for the formation of mSnO_2_/G. (**B**) The discharge capacities as a function of cycle numbers for the three samples at 0.1 C. (**A**,**B**) Reprinted with permission from Ref. [67], Copyright (2013) John Wiley & Sons. (**C**) TEM (**D**) HRTEM images of carbon-coated mSnO_2_/G/mSnO_2_. (**E**) Rate performance of carbon-coated mSnO_2_/G/mSnO_2_. (**C**–**E**) Reprinted with permission from Ref. [76], Copyright (2019) Elsevier. (**F**) Schematic representation showing paths for Li ions and electrons in the (**G**) Charge/discharge curves of sandwich-like N-doped SnO_2_/G. (**F**,**G**) Reprinted with permission from Ref. [92], Copyright (2012) John Wiley and Sons.

The high-capacity alloying (Sn, Si, Ge) or conversion (Co_3_O_4_, NiO) materials have been combined with G nanosheets. Among them, SnO_2_ has been most extensively investigated in preparation of G-based mesoporous materials due to the well-established sol-gel synthesis of SnO_2_ clusters suitable for self-assembly with GO nanosheets. Generally, SnO_2_ suffers from significant volume change during the conversion to Sn and subsequent lithium (de)alloying processes, which lead to rapid capacity fading and severe aggregation of Sn clusters [154,155]. To alleviate this problem, SnO_2_ was dispersed on the G surface, offering extra space to regulate the volume change and avoiding the aggregation of Sn clusters. Yang et al. synthesized mSnO_2_/G with high specific surface area of 251.7 m^2^ g^−1^ and pore size of 3.8 nm, and evaluated its electrochemical performance as an anode for LIB (Figure 12A,B) [67]. For the typical SnO_2_-based anodes, the high plateaus were usually observed because of the transformation of SnO_2_ + Li^+^ to Sn + Li_2_O to produce SEI layers which leads to low CE and discharge capacity at the first cycle. However, in the case of mSnO_2_/G, the high plateau almost disappeared after the first cycle, and the conversion of Sn to Li_x_Sn alloy occurred reversibly. Since G improved the electronic conductivity of the electrode and mitigated the transformation from the volume change, mSnO_2_/G exhibited a high CE of 69.4% at the first cycle, afterward above 95.3% at the following cycles. Additionally, the mesopore of electrodes provided a large specific surface area to accommodate higher amounts of Li^+^ ions, enhancing the reversible capacity. Even at a high current density of 782 mA g^−1^, mSnO_2_/G showed stable reversible capacity of 621.5 mAh g^−1^. Moreover, SnO_2_ NPs dispersed on the G surface were used as anodes of LIBs. In 2009, nanoporous electrode materials were fabricated by synthesizing G nanosheets decorated with SnO_2_ NPs (SnO_2_/G) [85]. Since G nanosheets were homogeneously distributed between loosely packed SnO_2_ NPs, the SnO_2_/G had free space to buffer volume change. Thus, SnO_2_/G showed high capacity of 570 mAh g^−1^ without electrode pulverization.

In 2019, Yao et al. synthesized a carbon-coated mSnO_2_/G/mSnO_2_ electrode for LIB and evaluated the electrochemical performances (Figure 12C–E) [76]. Carbon-coated mSnO_2_/G/mSnO_2_ not only provided the free space to prevent agglomeration of SnO_2_ due to the uniform mesopores, but also hindered the exposure of active material to the electrolyte by carbon coating which could prevent the aggregation of MO NPs. Since nitrogen doping has been reported to enhance electrochemical performance of electrodes [156,157], they used the dopamine as carbon and nitrogen source. It contained a pyridinic N atom that accommodates more Li^+^ ions. As a result, carbon-coated mSnO_2_/G/mSnO_2_ electrode displayed high reversible capability of 1211 mAh g^−1^ after 300 cycles at 0.2 A g^−1^. Notably, at high current density of 10 A g^−1^, the reversible capacity reached a maximum of 315 mAh g^−1^. Similarly, Feng’s group synthesized the carbon coated SnO_2_/G (C-SnO_2_/G) and demonstrated excellent cyclability capable of delivering specific capacity (~800 mAh g^−1^) after 100 cycles [91]. The same group prepared G-based TiO_2_/SnO_2_ hybrid nanosheets (TiO_2_/SnO_2_-G) with uniform staggered distribution of rutile TiO_2_ and SnO_2_ nanocrystals [88]. Since the exposed SnO_2_ was spatially segregated by TiO_2_, agglomeration of SnO_2_ could be significantly suppressed. As a result, TiO_2_/SnO_2_-G maintained a reversible capacity of 600 mAh g^−1^ even after 300 cycles. Furthermore, the formation of Sn-N bonds between SnO_2_ and G suppressed the aggregation of Sn NPs during the discharging process, resulting in a large capacity, high-rate capability and excellent cycling stability of N-doped SnO_2_/rGO. For instance, Zhou et al. synthesized N-doped SnO_2_/rGO with reversible charge capacity of 1346 mAh g^−1^ after 500 cycles [89].

Fabrication of the sandwich structure is a promising strategy to enhance the electrochemical performance of SnO_2_/G composites. For non-sandwich G composites, due to the weak contact between the particle and G, the alloy agglomerates were easily peeled off from the G surface when cycling, resulting in rapid capacity fading. Wang et al. prepared sandwich-like N-doped SnO_2_/G as anodes for LIB (Figure 12F–G) [92]. Benefiting from the sandwich structure of SnO_2_/G, the CE rapidly increased to nearly 100% after the first cycle. Even at a current density of 5000 mA g^−1^, the capacity of sandwich-like N-doped SnO_2_/G electrodes was 504 mAh g^−1^, which is higher than that of graphite electrodes (372 mAh g^−1^). As an anode for LIBs, the sandwich structure could provide a continuous conductive path between the SnO_2_ nanocrystals, reducing the particle–particle interface resistance. Inspired by this, a variety of sandwich-like G-based MO (e.g., CO_3_O_4_, NiO) were prepared. Since CO_3_O_4_ and NiO had a good theoretical capacity of 892 and 718 mAh g^−1^, respectively, they could be excellent candidates for G-based MO. Yang group prepared sandwich-structural G-based CO_3_O_4_ (CO_3_O_4_/G) and NiO (NiO/G) as anodes for LIB. Compared to normal G-based CO_3_O_4_ and NiO, the CO_3_O_4_/G and NiO/G showed improved specific capacity of 310 mA g^−1^ and 718 mA g^−1^ at 1 C, respectively [94].

## 4. Conclusions

We have discussed the synthesis strategies for G-based 2D mesoporous materials and summarized their structural advantages in SCs and LIBs. The surface chemistry of GO and inorganic precursors has been tailored to achieve controllable growth of inorganic mesoporous layers on the GO surface. Thus, the surface modification of GO such as oxidation, carboxylation, amination, heteroatom doping, polymer coating is frequently conducted. Additional bridging molecules (amino acids, glucose) are sometimes utilized to increase the interaction between GO and inorganics. Furthermore, pH of the solution was adjusted to induce favorable electrostatic interaction between GO and other components. In addition, controlled sol-gel process has been applied to deposit inorganic MOs selectively on the functional groups of GO. Despite the considerable progress made so far, there are still several challenges to be addressed.

From the materials synthesis point of view, large-scale production of high-quality G is the major problem to be resolved for the widespread use of G and G-based mesoporous materials. Although several scalable syntheses of G have been reported, the mass-produced G usually shows poorer performance compared with the best samples obtained in research laboratories [12,109,158,159]. Subsequent deposition of mesoporous inorganic materials and various chemical/thermal treatments make industrial mass production even more difficult. Alternatively, the use of other 2D materials as substrates [160,161,162,163] or layer-structured materials as templates [164,165] have been reported, however they still have the same problems. In this regard, one-step bottom-up synthesis of porous carbon and porous carbon/inorganic composites has attracted great attention [166,167,168,169]. Direct transformation of inexpensive resources to G-like porous materials would be also a possible solution to the mass production problem [170,171]. In addition, the currently used methods are usually applicable to a few compositions (silica, carbon, or TiO_2_ and SnO_2_) or greatly lack the ability to control pore size and structures. Since the intrinsic properties of 2D materials are highly dependent on their structural features, the development of an efficient synthesis method that enables the precise control over composition, aspect ratio, thickness, degree of graphitization, pore size and structures will be of particular research interest.

From the energy storage application point of view, there are several issues to be addressed in G-based 2D mesoporous materials. When used as anodes in LIBs, the large surface area of G often results in poor initial CE and large voltage hysteresis upon lithiation/delithiation. A considerable quantity of Li^+^ ions were irreversibly consumed to form SEI during the first lithiation step. Li^+^ ions can be also irreversibly stored on defects such as oxygen- and/or hydrogen-containing surface groups. In this respect, pre-lithiation, and removal of undesired surface groups are possible solutions [172,173]. Coating G with a low-surface-area mesoporous material can also minimize the exposed surface area. Similarly, the 3D morphology engineering of 2D nanosheets can lower the electrolyte decomposition reactions by decreasing the outermost surface area [174]. On the other hand, in EDLCs, the high specific surface area of G-based mesoporous materials is highly advantageous to increase the energy density because EDLCs store the energy through electrostatic adsorption of ions on the surface of electrodes. Further introduction of pseudocapacitive active materials on G can increase the energy density of SCs. However, it still remains challenging to prepare SC electrodes with high energy and power density simultaneously. In this regard, engineering the morphology and pore structures of G-based materials will play vital roles in improving the performance of SCs. In particular, G-based 2D materials with well-defined interconnected hierarchical pore structures are excellent candidates due to their combined advantages of 2D morphology (high packing density and volumetric capacitance) and multi-dimensional pore structures (high ion adsorption, fast ion transport, good electrolyte permeability). Finally, benefitting from 2D morphology with large lateral size and atomic thickness, G-based 2D mesoporous materials are particularly attractive for the preparation of flexible electrodes for LIBs and SCs, which will be the intensive research topic in the future [37,175,176].

## Figures and Tables

**Table 1:** Summary of graphene-based two-dimensional (2D) mesoporous materials.

Synthesis Strategies	Composition	Surface Area (m^2^ g^−1^)	Pore Size (nm)	Applications	Ref
Soft templating	mSiO_2_/G	980	2	Lithium ion battery (LIB)	[55]
		925	4.2	N/A	[56]
		927	2.7	Biphasic catalysis	[57]
		806	3.7, 5.4	N/A	[58]
		1252	2.5	Biomedical application	[59]
	mSiO_2_- Pt/G	1057	2.8	Oxygen reduction reaction (ORR)	[60]
	mSiO_2_-PEI/G	930	0.7–2.0	CO_2_ capture	[61]
	mC/G	903	10.7	Supercapacitor (SC)	[62]
	N-doped mC/G	414–422	8–9	ORR	[63]
	N-doped mC-Fe/rGO	968.3	N/A	ORR	[64]
	N-doped mC-Mo_2_C/rGO	389	14	Hydrogen evolution reaction (HER)	[65]
	N-doped mC-Fe_2_O_3_/rGO	373	12	ORR	[66]
	mSnO_2_/G	251.7	3.8	LIB	[67]
	TiO_2_/G	N/A	N/A	LIB	[68]
	mTiO_2_/G	150	6.5	N/A	[69]
	mSnPi/GO	170	7.4	ORR	[70]
Hard templating	mCN/G	542	<5	ORR	[71]
	N-doped mC/G	589	22	ORR	[72]
		832	13.9	SC	[73]
	mC/G	1179	~4	SC	[74]
	mTiO_2_/G	202	N/A	LIB	[75]
	mSnO_2_/G/mSnO_2_	385	3–4	LIB	[76]
	Nb_2_O_5_/G	158	3–20	Sodium ion battery	[77]
Template free	mC/GO	272	3–5	ORR	[78]
	N-doped mC/G	911	N/A	ORR	[79]
		256	~2	ORR and oxygen evolution reaction (OER)	[80]
		399	10.2	SC	[81]
	C/G	1293	0.8	SC	[82]
		1086	0.6	CO_2_ capture	[83]
	B/N-co-doped mC/G	363	17.1	ORR	[84]
	SnO_2_/G	N/A	N/A	LIB	[85]
	SnS_2_/C	N/A	N/A	LIB	[86]
	G/Sn/G	90.7	N/A	LIB	[87]
	TiO_2_-SnO_2_/G	138	3	LIB	[88]
	N-doped SnO_2_/rGO	N/A	2.2	LIB	[89]
	r(SG/GN)	130	5	LIB	[90]
	C-SnO_2_/G	N/A	N/A	LIB	[91]
	N-doped SnO_2_/G	N/A	N/A	LIB	[92]
	TiO_2_-Quantum dots(QDs)/G	N/A	N/A	LIB	[93]
	Co_3_O_4_/G	N/A	N/A	LIB	[94]
	NiO/G	N/A	N/A	LIB	
	TiO_2_/G-aerogels	204	4–5	LIB	[95]
	TiO_2_/GO	229	N/A	LIB	[96]
		252	3.4	LIB	[97]
	Co_3_O_4_/N-rGO	186.8	4	Zinc air battery	[98]
	Carbon black/G	532–586	N/A	SC	[99]
Top down method	Microwave exfoliated GO (MEGO)	~3100	0.6–5	SC	[100,101,102]
	rHGO paper	15	7–600	SC	[103]
	G nanomeshes (GNMs)	810–1010	10-hundres of nanometers	Peroxidase catalysts	[104]
	Microwave (MW)-rGO	832.38–965.64	5–100	N/A	[105]
	Porous G nanosheets (PGNs)	1374	2.4	SC	[106]
	Hierarchical porous G aerogels (HPGA)	266.4–383.7	21–53	LIB	[107]
	Porous G nanosheets (PGNs)	323.3–689	4–50	LIB	[108]
	N-doped porous G	400	20–50	N/A	[109]
	Holey GO (HGO)	430	2–3	SC	[110]
	rGO	N/A	N/A	Chemical sensing	[111]
	Holey G (HG)	658	N/A	SC	[112]
		447.5–733.5	N/A	Electro-oxidation of MeOH	[113]

**Table 2:** The G-based 2D mesoporous materials for supercapacitor (SC) applications.

	Composition	Capacitance [F g^−1^]	Cycling Stability	Ref.
SCs	mC/G by KIT-6	276 at 1 A g^−1^	97% of initial capacitance after 8000 cycles at 30 A g^−1^	[74]
	Carbon black(CB)/G nanosheet	175 at 10mV s^−1^	90.9% of initial capacitance after 6000 cycles at 200 mV s^−1^	[99]
	Activated MEGO	172 at 1 A g^−1^	97% of initial capacitance was maintained after 10,000 cycles at 2.5 A g^−1^	[100]
	Dual-mesoporous polypyrole/G (DM-PG)	376 at 1 mV s^−1^	94% of initial capacitance after 3000 cycles at 1 mV s^−1^	[133]
	Single-mesoporous PG (SM-PG)	332 at 1 mV s^−1^		
	self-assembled G hydrogel (SGH)	175 at 10 mV s^−1^	N/A	[134]
	Macroporous nitrogen-doped G hydrogel (GN-GH)	190 at 10 mV s^−1^	95.2% of initial capacitance after 4000 cycles at 100 A g^−1^	[135]
Na ion-hybrids SCs	mNb_2_O_5_/G	293 mA h g-1 at 50 mA g^−1^	N/A	[136]

**Table 3:** The G-based 2D mesoporous materials for lithium-ion battery (LIB) applications.

Composition	Reversible Capacity [mAh g^−1^]	Cycling Stability [mAh g^−1^]	Rate Capability [mAh g^−1^]	Ref.
mC/G	915 at 0.2 C	770 after 30 cycles at 0.2 C	370 at 5 C	[55]
3D hierarchical porous G aerogels (HPGA)	1100 at 0.1 A g^−1^	1100 after 100 cycles at 0.1 A g^−1^	300 at 20 A g^−1^	[107]
TiO_2_ NPs on a G aerogels	202 at 0.59 C	200 after 50 cycles at 0.59 C	99 at 5 A g^−1^	[95]
TiO_2_ QDs/G	190 at 1 C	190 after 100 cycles at 1 C	101 at 50 C	[93]
TiO_2_/G	162 at 1 C	200 after 30 cycles at 0.2 C	80 at 50 C	[75]
	94 at 10 A g^−1^	175 after 100 cycles at 0.59 C	149 at 0.1 A g^−1^	[96]
	237 at 20 mA g^−1^	237 after 100 cycles at 20 mA g^−1^	247 at 0.1 C	[97]
SnO_2_/G	621.5 at 782 mA g^−1^	916.9 after 30 cycles at 0.1 C	800 at 0.5 C	[67]
	810 at 50 mA g^−1^	570 after 30 cycles at 50 mA g^−1^	N/A	[85]
mSnO_2_/G/mSnO_2_	1211 at 0.2 A g^−1^	703 after 1200 cycles at 1 A g^−1^	315 at 10 A g^−1^	[76]
C-SnO_2_/G	800 at 200 A g^−1^	800 after 100 cycles at 200 A g^−1^	260 at 5 A g^−1^	[91]
TiO_2_/SnO_2_-G	600 at 160 mA g^−1^	600 after 300 cycles at 160 mA g^−1^	260 at 4 A g^−1^	[88]
N-doped SnO_2_/rGO	1352 at 0.5 A g^−1^	1346 after 500 cycles at 0.5 A g^−1^	417 at 20 A g^−1^	[89]
N-doped SnO_2_/G	918 at 100 mA g^−1^	910 after 100 cycles at 100 mA g^−1^	504 at 5 A g^−1^	[92]
CO_3_O_4_/G	680 at 0.1 C	715 after 50 cycles at 0.1 C	310 at 1 C	[94]
NiO/G	660 at 0.1 C	617.6 after 50 cycles at 0.1 C	718 at 1 C	

## Data Availability

No new data were created or analyzed in this study. Data sharing is not applicable to this article.
